# Body odours as a chemosignal in the mother–child relationship: new insights based on an human leucocyte antigen-genotyped family cohort

**DOI:** 10.1098/rstb.2019.0266

**Published:** 2020-04-20

**Authors:** Laura Schäfer, Agnieszka Sorokowska, Jürgen Sauter, Alexander H. Schmidt, Ilona Croy

**Affiliations:** 1 Department of Psychosomatics, Technische Universität, Fetscherstraße 74, 01307 Dresden, Germany; 2 Smell and Taste Lab, Department of Psychology, University of Wroclaw, pl. Dawida 1, 50-527, Wroclaw, Poland; 3 DKMS, Kressbach 1, 72072 Tübingen, Germany; 4 DKMS Life Science Lab, St. Petersburger Straße 2, 01069 Dresden, Germany

**Keywords:** olfaction, parent–child relationship, body odour, major histocompatibility complex, hormones, chemosensory communication

## Abstract

Mothers are able to identify the body odour (BO) of their own child and prefer this smell above other BOs. It has hence been assumed that the infantile BO functions as a chemosignal promoting targeted parental care. We tested this hypothesis and examined whether children's BOs signal genetic similarity and developmental status to mothers. In addition, we assessed whether BOs facilitate inbreeding avoidance (Westermarck effect). In a cross-sectional design, *N* = 164 mothers participated with their biological children (*N* = 226 children, aged 0–18 years) and evaluated BO probes of their own and four other, sex-matched children. Those varied in age and in genetic similarity, which was assessed by human leucocyte antigen profiling. The study showed not only that mothers identified and preferred their own child's BO, but also that genetic similarity and developmental status are transcribed in BOs. Accordingly, maternal preference of their own child's odour changes throughout development. Our data partly supported the Westermarck effect: mothers' preference of pubertal boys' BOs was negatively related to testosterone for the own son, but not for unfamiliar children.

This article is part of the Theo Murphy meeting issue ‘Olfactory communication in humans’.

## 1. Introduction

An affective tight bond between a primary carer and their child provides essential physical and psychological safety, thus ensuring healthy development of the child [1]. The formation of such a bond can be attributed to a large investment of time, emotions, finances and sociability from the carer. The carer's motivation for such investment is facilitated by compelling characteristics of their child, including infantile visual or auditory cues. For instance, a child's relatively large eyes, small mouth and round face, as described in the Kindchenschema [2], constitute an infantile cue that promotes gentle and social behaviour in the carer (for a review, see [3]) by coding pleasure, motivation and reward in the parental brain [4].

Another potential factor affecting parental investment is genetic relatedness. On average, parents are willing to invest more resources in genetically related than in step-children [5], a behaviour that is likely facilitated by kin recognition. For example, a child's protruding ears might trigger visual kin recognition by means of resemblance and show a parent with relatively large ears that this child belongs to the same family. In fact, facial resemblance affects a parent's level of investment in their child, especially for fathers [6].

Chemosignalling as a cue for parental investment has scarcely been investigated, but the few existing works show that body odour (BO) similarity can increase parental investments by fathers [7]. BO recognition is a significant predictor of positive mothering attitudes and nursing experiences [8] and relates to higher emotional closeness for fathers and lower rate of corporal punishment for mothers [9]. Another group of studies revealed increased preference of odours from close kin and BO identification abilities within families [9,10]. BO preference is mediated by identification ability; in contrast to healthy mothers who are able to identify their own child's odour shortly after birth [10], mothers with impaired bonding to their child can neither identify nor prefer their own child's BO [11]. Whereas the maternal ability is preserved irrespective of whether the child is infantile, pubertal or post-pubertal, there are inconsistencies in paternal olfactory kin recognition across these developmental stages [10,12,13].

A carer's evaluation of their own child's BO changes over time. Parents perceive the BO of infants aged younger than 12 months as pleasant [8] and the odour of their own infant is preferred over those of other infants. Yet, biological cues that support the development of a tight bond between mother and child become less important with increasing age of the child (as in facial characteristics [14]). Likewise, the preference for one's own offspring's odour seems to decline as a function of development, as suggested by two recent questionnaire studies. One revealed that the carer's regard for their own child̀s BO continuously decreases between the age of 3 years and adolescence [15], while another showed that mothers of children below 3 years and fathers of children below 1 year old report more frequent intentional affectionate sniffing of their child's BO than parents of older children [16].

The two existing experimental studies that examined a parent's evaluation of their own child's BO in pre- pubertal and pubertal stages have revealed conflicting results. Weisfeld *et al.* [13] found in a sample of 21 families that fathers dislike the odour of their pubertal daughters. This study was interpreted in line with the so-called ‘Westermarck effect’, which describes the lack of sexual attraction between adults who cohabited as children [17,18]. The aversion to familiar, opposite-sex BOs may hence serve as an olfactory mechanism for preventing inbreeding. However, this result was not replicated in a later sample of nine families [12].

BO comprises genetic, developmental and environmental compounds. The latter refers to highly variable environmental influences such as food, hygiene, disease or culture [19]. Developmental compounds are also variable as they change throughout development: the onset of puberty coincides with a rise in steroid hormones including testosterone and oestradiol [20]. Steroid hormones reflect immunological functioning and are therefore considered to signal genetic health of a potential mate [21]. They are not directly responsible for BO composition but transformation of those steroids via skin bacteria leads to an olfactory percept [22]. Testosterone has been suggested to affect BO ratings [23], e.g. in terms of a more intense and more negative evaluation [24,25], but evidence is mixed [26]. Oestradiol was shown to influence olfactory transmitted sexual attraction in mice [27] and facial attractiveness in human [28] but whether it similarly affects human BO ratings has been unclear. Female assessment of male BOs does not seem related to oestradiol concentration [26]. However, female BOs change around the menstrual cycle and are rated as most pleasant by men around ovulation [19], when oestradiol levels peak [29]. It is unknown whether oestradiol concentration mediates the perceived change in BO [19]. Male ratings of women's BO attractiveness is predicted by the women's oestradiol level [30].

By contrast, olfactory kin recognition is presumably based on stable compounds of BO, which may be genetically influenced [31]. Adult relatives, as well as children, are recognizable via BO [32,33], so odour-mediated kin recognition may also be important beyond childhood. By excluding relatives, kin recognition helps identify potential mates while avoiding potential disadvantages of inbreeding, such as a reduced offspring fitness [34]. A well-researched part of the genetic profile that impacts BO assessment is the human leucocyte antigen (HLA) system (or major histocompatibility complex, MHC, in animals). The HLA is involved in the body's immune response by activating T cells through the presentation of peptides [35]. HLA molecules function as chemosignals [36] and partially account for genetic variance in BO. Although the interaction of phenotypic and genetic traits in olfactory kin recognition is not yet well understood, it is known that HLA relates to BO perception. Women prefer the BO of men whose HLA system is different (to a certain degree) from their own [37]. On the other hand, people prefer their own odour when it is supplemented by self rather than non-self MHC ligands [38]. Thus, in a non-mating context, this ability may contribute to parental olfactory identification and preference of the own child's BO, especially in ambiguous situations lacking clear indicative cues (e.g. distinguishing between children of the same age with similar hormonal levels).

These lines of evidence suggest that BOs may promote bonding and incest avoidance by triggering an evaluation incorporating genetic (HLA, sex), developmental (e.g. age, hormonal status) and environmental factors. However, previous results on BO perception in the parent–child dyad are mixed and based on small sample sizes. We therefore aimed to examine maternal perception of children's BO during development—from infanthood to adolescence. We hypothesized that (H1) mothers are able to identify their own child's BO and that this identification relates to genetic (HLA) similarity; (H2) mothers prefer the BO of their own child over the BO of other children and that this preference relates to HLA similarity; (H3) preference for the own child̀s BO declines after infanthood; (H4) assessed pleasantness of BOs is higher for those probes identified as one's own child. Furthermore, we expect an additional effect during puberty, namely that (H5) the assessed pleasantness for the BO of one's own opposite-sex child (here, sons) decreases as sons transition across puberty, whereas this preference remains stable for the same-sex child (daughters) and for unfamiliar children.

## 2. Method

The ethics committee of the University of Dresden (Code: EK 104032015) approved the conduction of the study in accordance with the ‘World Medical Association's Declaration of Helsinki’. Written, informed consent was obtained from all participants.

### (a) Participants

The sample included 164 normosmic mothers (*M* = 37.5, s.d. = 7.8 years) participating with at least one biological child (*M* = 7.6, s.d. = 5.9 years, *n* = 124 girls, *n* = 102 boys). The children were grouped into four age groups, representing stages of hormonal development [20]: infants (*n* = 38 girls and 39 boys; aged 0–3 years, *M* = 1.18, s.d. = 0.96), pre-pubertal (*n* = 30 girls and 23 boys; aged 4–8 years, *M* = 5.96, s.d. = 1.37), pubertal (*n* = 23 girls and 21 boys; aged 9–13 years, *M* = 10.91, s.d. *=* 1.56) and post-pubertal (*n* = 33 girls and 19 boys; aged 14–18 years, *M* = 16.00, s.d. = 1.24). Post-pubertal therefore does not imply that development is completed, but rather that the phase of hormonal changes and main stages of puberty have begun; whereas pubertal status implies that the phase of hormonal changes and main stages of puberty are about to begin. In order to underpin the definition of these categories, we additionally assessed pubertal status by hormonal analyses and the Pubertal Development Scale (PDS, [39], see below). The four age groups were used to match the BO samples and for further analyses (see below). Demographic variables and descriptive statistics for all measures are listed in electronic supplementary material, tables S1 and S2.

#### (i) Inclusion and exclusion criteria

Biological parenthood of at least one child between 0 and 18 years was required for inclusion in the study. Exclusion criteria included insufficient German language skills, pregnancy and chronic disease or disability of the child. Anosmia and hyposmia also served as exclusion criteria, this being tested with a screening version of the standardized Sniffin’ Sticks Step II**®** (Burghart, Wedel, Germany). Participants had to identify three odours (cinnamon, banana, fish odour) in a 4-alternative forced-choice task. The application of this test is sufficient to assess normosmia, ensuring reliable results with a sensitivity of 80.4% and specificity of 84.3% [40]. If the participant could not identify all the three odours correctly, the full identification subtest consisting of 16 odours was performed and participants were excluded if they failed to correctly identify at least 12 [40]. In addition, participants were asked if they currently suffer from smell problems (e.g. rhinitis) before starting the experiment in session 2. If the answer was affirmative, they were asked to attend a later appointment after recovery.

In total, 202 families were recruited for this study, of which two were excluded because mothers became pregnant before the second appointment. Thirty-six further families dropped out before the second session because of a ‘lack of time’ (26 families), ‘moving to another city’ (two families) or unspecified reasons (eight families). We also attempted to recruit fathers, but these data are not presented as the sample size was insufficient.

### (b) Procedure

Participants were recruited with flyers and advertisement among employees of the university hospital, in schools and kindergartens, as well as with personal invitations. After an initial contact was made, inclusion and exclusion criteria were announced via e-mail.

#### (i) First session

In the first session, mothers and children came to our Laboratory of the Department of Psychosomatics at the University Hospital, Dresden. The general procedure was explained and exclusion and inclusion criteria were verified. Besides tests of olfactory performance, the mothers completed medical history, demographic and standardized questionnaires (see §2g) and the HLA sampling was done (see §2c). Afterwards, the subjects received the study kit containing all utensils for taking hormone and BO samples at home, detailed oral and written instructions, and a questionnaire to complete after BO sampling (see §2e). This contained questions about the situation at home (smoking inside the home, pets, number of persons in the room where the child sleeps), the child's medical condition (drug use, e.g. antibiotics; illness) and contamination of the BO sample (e.g. by urine or faeces) on the experimental night. The first session lasted about 30 min.

The samples and the study protocol were collected the following morning. The samples (t-shirt/onesie) were then cut in half and frozen by −25°C for up to eight weeks before the second session [41]. For the onesies worn by younger children, only the upper part was used to avoid contamination with other odours (e.g. urine).

#### (ii) Second session

Only the mothers came to the second study session. They were instructed to refrain from wearing perfume on the test day, and not to eat or drink coffee 1 h prior to the experiment. Participants completed additional questions about current pregnancy, the day of their menstrual cycle and current use of hormonal contraception. After explaining the procedure, odour assessment took place.

The second session took between 45 and 60 min depending on the number of children of the mother.

### (c) Human leucocyte antigen sampling

Mothers and children were instructed to rub the inner skin of each cheek for 60 s with one buccal swab on each side. The study assistant did the sampling of babies. The buccal swabs were then dried for 5 min and stored in an envelope until genetic analysis. The samples were sent to the ASHI-accredited DKMS Life Science Lab (Dresden, Germany) for HLA analysis, which was done based on high-resolution typing including determination of the nucleotide sequence of exons 2 and 3 of HLA-A, -B, -C, -DRB1, -DQB1 and -DPB1 with next-generation sequencing [42,43].

### (d) Developmental status

Developmental status was assessed for all children above the age of 4 years. Children were asked to provide an additional saliva sample in order to determine pubertal status via hormonal analysis. For that purpose, they were equipped with a salivette (Salivette®, code blue, SARSTEDT AG & Co. KG, Nümbrecht, Germany) and parents were instructed to explain to the children that they had to chew for 60 s on the salivette until the cotton contained sufficient saliva for laboratory analysis. In order to assess the relationship between BO assessment and levels of steroid hormones, saliva samples were collected in the evening immediately before the test night.

After sampling, the salivette was stored overnight in the fridge and brought back to the laboratory the next morning together with the BO samples. Saliva samples were frozen (−25°C) and then sent to the Dresden LabService GmbH, where the concentrations of testosterone and oestradiol were determined by immuno-assay analyses.

Furthermore, mothers were asked to rate the children's pubertal status with the PDS [39]. The PDS contains three questions asking about development of body hair, growth of breast/beard, menarche and voice break and is characterized for each sex with a global score ranging from 0 (puberty has not begun) to 12 (development completed). Evaluation of the German version of the PDS shows acceptable reliability (*r* = 0.64 − 0.69) and validity (self- versus external assessment, *r* = 0.39 and 0.83; [39]).

### (e) Body odour sampling

Parents collected BO samples at home by following a standardized protocol: in the first session, the families received a study kit containing an appropriately sized 100% cotton t-shirt/onesie for each child, which had been recently washed with odourless detergent (Denkmit Vollwaschmittel Ultra Sensitive, dm-drogerie markt GmbH & Co. KG, Karlsruhe, Germany, www.dm.de). In addition, the mothers were provided with the same odourless detergent, an odourless medical shower gel (both EUBOS flüssig wasch + dusch, Dr. Hobein GmbH, Meckenheim, Germany, www.eubos.de), a re-sealable plastic zip-bag and the questionnaire concerning their conformity with the instructions, which the mothers had to fill in before and after the experimental night. Mothers were instructed to use the odourless detergent to wash any clothes worn in addition to the sample shirt, as well as the child's bedsheets, prior to the experimental night. Further, mothers were instructed to wash their children with the odourless shower gel in the evening and to refrain from using any perfumed hygiene products. On the following morning, the shirt/onesie was stored in the plastic zip-bag and delivered to the laboratory. When delivery would take longer than 2 h, subjects were instructed to cool the BO sample until it was brought to the laboratory.

### (f) Experiment: body odour matching and rating

#### (i) Body odour matching

**Figure 1.**
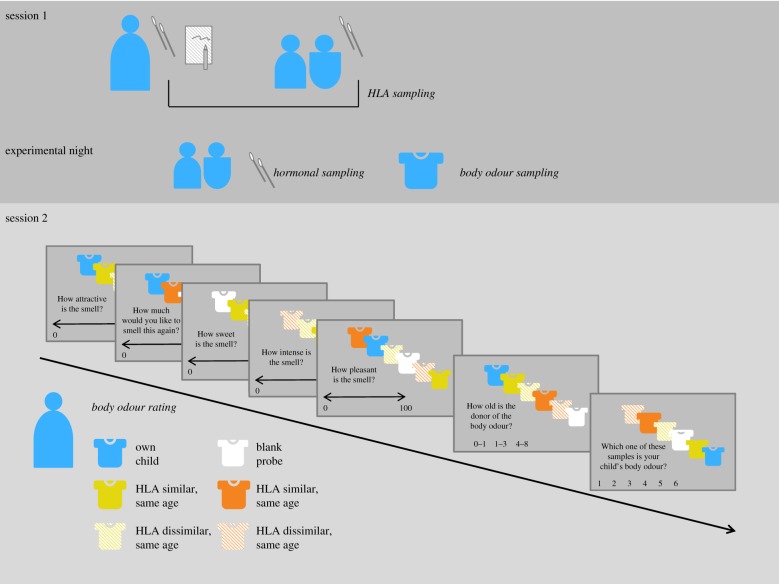
Study design and procedure. Session 1, sampling procedure; session 2, experiment with rating procedure.

Prior to the experiment, the mothers' HLA profiles were matched to the HLA of four unfamiliar children (all sex-matched to the own child). Each mother was matched to two unfamiliar children of the same age group as their own child (one HLA-similar and one HLA-dissimilar child) and to two of a different age group from their own child (again, each one HLA-similar and one HLA-dissimilar child, both equivalently older or younger than the mother's own child; figure 1). HLA similarity was defined as a match in at least one allele in the loci HLA-B and/or -C (similar = 1–4 matches; see electronic supplementary material, supplementary information on BO matching), while HLA dissimilarity was defined as no matches at HLA-B and/or -C, in accordance with previous studies [44,45]. In the statistical analysis, the HLA influence is referred to as ‘BO probe’.

#### (ii) Body odour rating

Participants had to rate six BO probes: the BO of their own child and four additional, sex-matched BO samples (see above), as well as a blank probe (an unworn *t*-shirt washed beforehand with the same odourless detergent and stored in a plastic zip-bag). The blank probe was used as a control for sufficient intensity of the BO samples.

The samples used for the trial were defrosted 1.5 h before starting the experiment. The study assistant wore odourless rubber gloves and refrained from wearing perfume in order to avoid biasing the odour perception of the participant. First, we conducted a test trial, in which each probe was presented without any assessment in order to ensure anchoring of the stimuli intensity. Afterwards, each BO sample was presented seven times in randomized sequence (5× hedonic ratings, 1× age group rating, 1× identification of the own child, figure 1) and after each presentation, participants assessed one of a total of seven ratings.

At the beginning of each trial, the study assistant instructed the mother to close her eyes during 6 s of smelling. Then, the study assistant held each sample for 6 s with the armpit part upwards directly under the nose of the subject. Each sample was placed back after the mother had smelled it, and she was then instructed to open her eyes and to rate the stimulus. Closing the eyes facilitated concentration and hindered mothers from seeing if the sample was part of a *t*-shirt or of an onesie, which would provide information on the age group of the child.

The ratings included five hedonic (pleasantness, intensity, reward value (wanting), sweetness, attractiveness) and two identification dimensions (identification of the respective age group of the BO donor; identification of the own child's BO, figure 1). Ratings were carried out using visual analogue scales (VAS) ranging from 0 (‘very unpleasant’, ‘not intense at all’, ‘I don't want to smell this again at all’, ‘not sweet at all’, ‘not attractive at all’) to 100 (‘very pleasant’, ‘very intense’, ‘I would love to smell this odour again’, ‘very sweet’, ‘very attractive’). For age group assessment, the subjects had to choose one out of six age categories: less than 1 year, 1–3 years, 4–8 years, 9–13 years, 14–18 years, greater than 18 years. In the last trial, mothers judged which of the BO samples belonged to their own child. This was the only trial in which the odours were presented twice when the subject asked for a repetition. Afterwards, a pleasant (orange) and an unpleasant odour (fish) were presented serving as odour controls for hedonics of olfactory ratings. On average, orange (*M* = 79.05, s.d. = 20.08) was significantly better rated than fish (*M* = 11.20, s.d. = 15.95; *t*_225_ = 43.79, *p* < 0.001).

### (g) Measures

#### (i) Sociodemographic status

To assess sociodemographic status, subjects were asked to state age, sex, immigration status, highest school-leaving qualification, professional qualification and degree, sexual orientation (hetero-, homo- or bisexual), relationship status (partnership with biological parent of the child, partnership with a new partner, no partnership), number of children, number of biological children, age and sex of each child.

#### (ii) Medical and psychological status

To assess medical status, subjects were asked to state disease status, alcohol and smoking status, exposure to gas/chemicals, and smell problems. In addition, mothers were asked to provide information about pregnancy or birth complications, preterm birth, disability or serious disease of the child.

***Depression score.*** As depression can affect olfactory performance (for a review, see [43]), this was controlled by the ‘Beck Depression Inventory II’ (BDI-II, [44]), a widely used questionnaire for evaluating the severity of depression.

***Behavioural Inhibition and Activation.*** In order to measure the mothers' general reward perception, the Behavioural Inhibition and Activation System Scale was assessed [45]. The questionnaire consists of 24 items with a 4-point scale (1 = strong disagreement, 4 = strong agreement), such as ‘When I get something I want, I feel excited and energized’. Scores are summed up to four subscales indicating Behavioural Inhibition System, Behavioural Activation System (BAS) Reward Responsiveness, BAS Drive and BAS Fun Seeking. The scales show sufficient internal consistency (*α* = 0.65–0.83 [46]).

Participants completed additional questionnaires on the mother–child relationship, which were not the focus of this study.

## 3. Statistical analyses

All data were analysed with IBM SPSS Statistics 25 (IBM Corp. Released 2017. IBM SPSS Statistics for Windows, Version 25.0. Armonk, NY: IBM Corp).

### (a) H1: mothers are able to identify their own child above chance and identification relates to human leucocyte antigen similarity

Here *χ*^2^-tests and binomial tests were used to compare the identification performance to the distribution by chance for each BO probe, and between and within the age groups.

#### (i) Identification across all children

A one-sample *χ*^2^-test assessed the overall observed distribution against the distribution expected by chance (1/6 = 16.7% for each BO probe). Subsequently, the distributions for each BO probe (own child, HLA-similar child same age, HLA-dissimilar child same age, HLA-similar child different age, HLA-dissimilar child different age) were assessed with binomial tests (versus chance level). Additionally, we tested whether mothers differed in identification performance depending on the sleeping environment (children sleeping in the same room versus in the same bed versus in a different room), but this did not influence identification performance ($\chi_{2}^{2}=1.84,$ *p* = 0.398).

#### (ii) Identification by age groups

The interaction effect between correct identification (yes versus no) and age group (0–3 years, 4–8 years, 9–13 years, 14–18 years) was analysed. For further exploration, binominal tests were calculated testing the observed distribution against the distribution expected by chance for each age group separately.

For all subsequent analyses, ratings of the blank BO sample were excluded to avoid overcomplicating the results and to focus on the hypotheses. The blank probe was perceived as significantly less intense than all other BO samples (*t*_225_ = 9.72, *p* < 0.001; intensity blank probe: *M* = 36.55, s.d. = 25.53; intensity averaged across BO samples: *M* = 53.88, s.d. = 14.65). For reasons of brevity, the results of the age ratings are not presented. *Pleasantness* ratings were chosen as the main outcome for analyses of hedonic assessment of the BOs, as this has been a common target in previous human olfactory studies investigating BO perception (e.g. [46]). In order to analyse maternal perception of the children's BOs, we used a generalized linear mixed model (GLM) with *pleasantness* as target of this analysis, in which each mother served as an individual, and each BO probe, as well as each child of the mother (in multiple-children families), served as a repeated measure. The respective effect of interest was then modelled as main and/or interaction effect, as described subsequently in detail for each hypothesis. For H2 and H3, we investigated BO probe and age group in one GLM; the assessed effects are listed below.

### (b) H2: Mothers prefer the body odour of their own child over other children and this preference relates to human leucocyte antigen similarity

#### (i) Preference across all children

Target: pleasantness; main effect of BO probe.

We further calculated a preference score by subtracting the pleasantness scores averaged across all unfamiliar BO probes from the score for the mother's own child's BO probe.

Using Bayesian statistics [47,48], we additionally explored the likelihood of the first parts of H1 (identification of the own child above chance) and H2 (preference of the own over other children), as for these hypotheses basic assumptions about the effect sizes of preference and identification of one's own child are available based on a previous publication [11]. For further information, see electronic supplementary material (Bayesian analyses).

### (c) H3: The preference of the own child's body odour declines after infanthood

#### (i) Preference by age groups

Target: pleasantness; main effect of age group of the child (1: 0–3 years, 2: 4–8 years; 3: 9–13 years; 4: 14–18 years); interaction effect between BO probe × age group.

Subsequently, the maternal preference score was tested within age groups (binomial test: preference score versus 0 (greater than 0 = preference)) in order to explore preferences to their own child's BO in each age group. In addition, we compared pleasantness ratings of the control odours (orange, fish) between the age groups with a repeated measures ANOVA controlling hedonic perception across the different age groups.

### (d) H4: The assessed pleasantness is higher for those body odour probes identified as the own child

In addition, we explored whether maternal pleasantness perception was related to the BO sample chosen as the ‘own child’ (chosen probe = yes/no, irrespective of false or correct choice).

#### (i) Pleasantness by identification

Target: pleasantness; main effect: chosen probe (yes/no); twofold interaction effects: BO probe × chosen probe, age group × chosen probe.

### (e) H5: The assessed pleasantness for the body odour of the own opposite-sex child (sons) decreases with increase in the sons' developmental status whereas the assessed pleasantness remains stable for the same-sex child (daughters)

For assessment of the hypothesized decline in maternal pleasantness ratings for their son's BO during development, we included only children above the age of 4 years (pre-pubertal, pubertal and post-pubertal age groups) in an ANOVA with the preference score as target. For each developmental marker (pubertal status (PDS score), hormonal status (testosterone, oestradiol)) and each sex, two models assessed the following interactions:

**Girls**: PDS score × age group; oestradiol × age group
**Boys**: PDS score × age group; testosterone × age group

In order to ensure robust distribution for statistical analyses, we log-transformed the hormonal data with lg(10).

**Post hoc tests.** For all models of H2–H4, pairwise post hoc comparisons were analysed using *t*-tests. Mean values (*M*) and 95% confidence intervals (reported as CI) are presented (electronic supplementary material, table S3). For all models of H5, Pearson correlation coefficients served for post hoc bivariate correlational analyses.

## 4. Results

### (a) H1: mothers are able to identify their own child above chance and identification relates to human leucocyte antigen similarity

#### (i) Identification across all children

**Figure 2.**
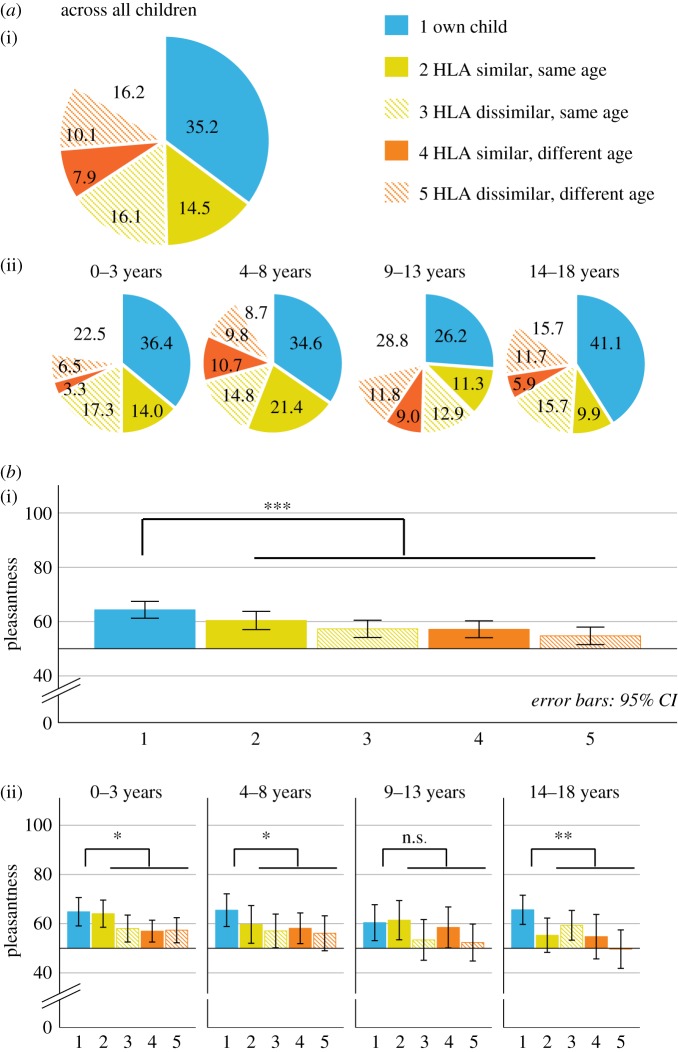
(*a*) Identification of BO probes depicted in % (i) across all children and (ii) for each age group. (*b*) Maternal preference of each BO probe and comparison between own child's BO versus all other samples depicted (i) across all children and (ii) for each age group. *p < 0.05; **p < 0.01, ***p < 0.001; n.s. = not significant (*p* > 0.05). Pleasantness is measured visual analogue scale, ranging from 0 (not pleasant at all) to 100(very pleasant).

The observed distribution of identified BOs differed significantly from the expected distribution by chance ($\chi_{224}^{2}=64.64,$ *p* < 0.001). The mothers were able to correctly identify the BO of their own child (identification rate: 35.2%; CI: 29–42%). This exceeded the chance level of 16.7% (*p* < 0.001, figure 2*a*).

The HLA-dissimilar, same aged child (16.1%, CI: 12–22%, *p* = 0.467) and the HLA-similar, same aged child (14.5%; CI: 11–20%, *p* = 0.257) were not chosen above chance and hence no influence of HLA similarity was observed. The identification rate of the blank probe did not exceed chance level (16.2%, CI: 12–22%, *p* = 0.345).

The responses for BOs of children whose age group differed from the one of the own child were below chance (HLA-dissimilar, different aged child: 10.1%, CI: 7–14%, *p* < 0.001; HLA-similar, different aged child: 7.9%, CI: 5–12%, *p* = 0.005, figure 2*a*).

#### (ii) Identification by age group

Mothers could correctly identify the BO of their own child in all age groups except puberty (infants: 36.4%, CI: 27–48%; pre-pubertal: 34.6%, CI: 24–49%; post-pubertal: 41.1%, CI: 29–54%; each *p* < 0.001). For the pubertal age group, the identification of the own child's BO did not differ significantly from chance level (26.2%, CI: 16–42%, *p* = 0.062, figure 2*a*). Bayesian analyses supported the alternative hypotheses (identification of the own child better than chance) over the null hypothesis for age groups of 0–3, 4–8 and 14–18 years. Neither the null nor the alternative hypotheses were supported for the age group of 9–13 years (see electronic supplementary material, table S4).

### (b) H2: Mothers prefer the body odour of their own child before other children and this preference relates to human leucocyte antigen similarity

#### (i) Preference across all children

A significant main effect of BO probe (*F*_4,359_ = 5.82*, p* < 0.001) demonstrated that the own child's BO was rated most pleasant. The mean preference of the own child's BO was 7.1 (CI: 3.86–10.34), higher than for all other probes (figure 2*b*; see electronic supplementary material, table S3). The next most pleasant odour was from the HLA-similar, same aged child and the rating for this BO and the own child's BO did not differ significantly (*p* = 0.068; compare electronic supplementary table S3). Bayesian statistics favoured the alternative hypotheses (significant preference of the own child's BO compared to unfamiliar children) over the null hypothesis for age groups 0–3, 4–8 and 14–18 years, and supported the rejection of the alternative hypotheses for the age group of 9–13 years (compare electronic supplementary material, table S5).

### (c) H3: The preference of the own child's body odour declines after infanthood

#### (i) Preference by age groups

There was neither a significant main effect of age (*F*_3,678_ = 1.64, *p* = 0.178) nor a significant interaction effect between age and BO probe on maternal pleasantness ratings (*F*_12,203_ = 0.81, *p* = 0.638). The preference for the own child̀s BO was hence above zero in almost all age groups (preference score for infants*: 7.62,* CI: 1.1–13.51; pre-pubertal: 9.20*;* CI: 2.59–15.80; post-pubertal: 8.74, CI: 2.53–14.96, *p* = 0.008 to 0.05). Similar to the identification results, we found no significant preference of the own child in the pubertal age group (1.61, CI: −7.19–8.93, *p* = 0.652; figure 2*b*, see electronic supplementary material, table S3).

In order to ensure that the reduced pleasantness perception in the pubertal age group was not based on a general reduced hedonic perception of the particular mothers of that age group, we compared pleasantness ratings of the control odours orange and fish and found that the hedonic perception did not differ between the age groups (*F*_3,220_ = 1.75, *p* = 0.158) and that none of the post hoc comparisons were significant.

### (d) H4: The assessed pleasantness is higher for those body odour probes identified as the own child

#### (i) Pleasantness by identification across all children

**Figure 3.**
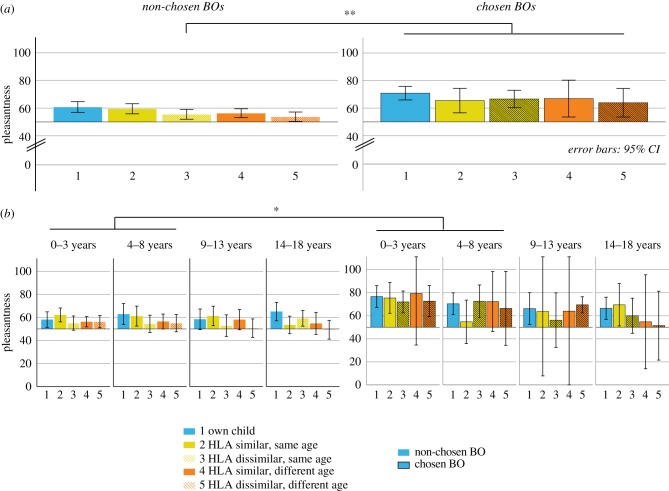
Maternal preference of BO probes depicted by identification behaviour: (*a*) left side: non-chosen BOs, right side: chosen BOs; (*b*) left side: non-chosen BOs depicted by age group; right side: chosen BOs depicted by each age group. *p < 0.05; **p < 0.01, ***p < 0.001. Pleasantness is measured visual analogue scale, ranging from 0 (not pleasant at all) to 100(very pleasant).

Mothers rated BO probes chosen as the ‘own child’ significantly more pleasant (*F*_1,805_ = 21.03, *p* < 0.001) compared to non-chosen BOs, irrespective of whether the choice was correct or false (figure 3*a*).

A significant interaction between BO probe and choice (*F*_8,492_ = 2.15, *p* = 0.030) further showed that BOs chosen as the own child were rated similarly pleasant (*F*_4,181_ = 0.64, *p* = 0.932), but BOs not chosen as the own child differed: among those non-chosen probes, pleasantness ratings of the own child and the HLA-similar same-aged child were significantly higher than ratings of the other BO probes (*F*_4,925_ = 2.49, *p* = 0.042), without any significant difference between the BO of the own child and the HLA-similar, same aged child, *p* = 0.641.

#### (ii) Pleasantness by identification in relation to age groups

The pleasantness ratings for chosen BOs were higher for BOs of pre-pubertal children (*F*_6,750_ = 2.29, *p* = 0.034): BOs (falsely and correctly) chosen as the own child's BO were rated as more pleasant in the infant and pre-pubertal age groups (0–3 years: 17.17, CI: 11.38–22.96, *p* < 0.001; 4–8 years: 8.69, CI: 1.45–15.90, *p* = 0.018) but not in pubertal and post-pubertal children (9–13 years: 6.37, CI: −1.47–14.13, *p* = 0.110; 14–18 years: 4.66, CI: −3.20–12.52, *p* = 0.244, figure 3*b*, see electronic supplementary material, table S3).

### (e) H5: The assessed pleasantness for the body odour of the own opposite-sex child (sons) decreases with increase in the sons' developmental status whereas the assessed pleasantness remains stable for the same-sex child (daughters)

#### (i) Relationship between developmental status and age

Pubertal status of boys correlated positively with the testosterone level (*r* = 0.50, *p* = 0.007); and likewise, girls with higher pubertal status exhibited increased oestradiol concentration, *r* = 0.33, *p* = 0.015. In older age groups, a higher level of pubertal status was observed for both boys (*r* = 0.77, *p* < 0.001) and girls (*r* = 0.87, *p* < 0.001). Older age was further associated with increased testosterone levels for boys (*r* = 0.51, *p* < 0.001) and with higher oestradiol levels for girls (*r* *=* 0.27, *p* *=* 0*.*026, electronic supplementary material, figure S1).

#### (ii) Influence of developmental status on maternal pleasantness ratings

Pubertal status did not relate to maternal preference (girls: pubertal status × age group: *F*_3,55_ = 0.67, *p* = 0.572; boys: pubertal status × age group: *F*_3,37_ = 0.22, *p* = 0.880).

**Figure 4.**
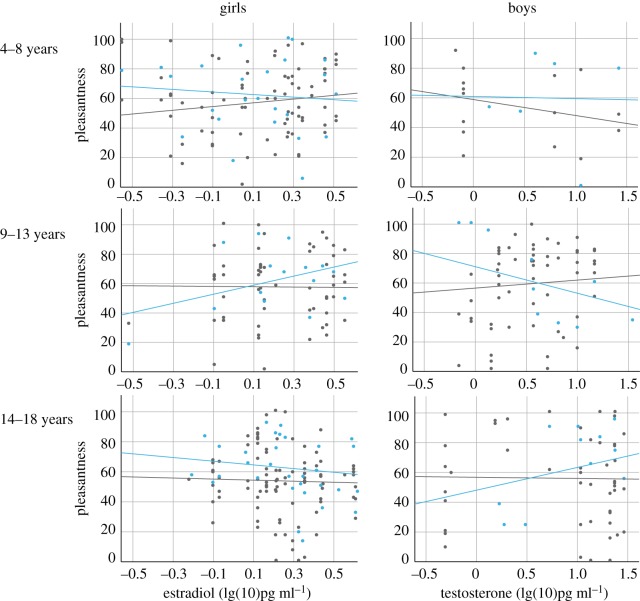
Scatterplots for maternal pleasantness ratings of BOs in relation to hormonal levels depicted by age group and sex of the child. Blue dots, own child; grey dots, unfamiliar child. Pleasantness is measured visual analogue scale, ranging from 0 (not pleasant at all) to 100(very pleasant).

For boys, a significant interaction between testosterone and age group emerged (*F*_3,41_ = 3.45, *p* = 0.026), but not between oestradiol and age group (*F*_3,41_ = 0.80, *p* = 0.502). Subsequent correlational analyses revealed a decrease in pleasantness ratings of own pubertal sons (age group 9–13 years) with elevated testosterone concentration (*r* = −0.53, *p* = 0.050, figure 4). No other significant correlations were observed. For girls, neither a significant effect of testosterone (*F*_3,60_ = 0.17, *p* = 0.918) nor of oestradiol concentration (*F*_3,71_ = 1.29, *p* = 0.254) on maternal pleasantness ratings was observed. Post hoc correlational analyses for each age group showed a positive association between pleasantness ratings of own pubertal daughters (age group 9–13 years) and oestradiol levels (*r* = 0.51, *p* = 0.041). Excluding the outliers (hormonal value of −0.50 or less; figure 4) from the sample, the correlation was no longer significant. No other significant correlations emerged.

## 5. Discussion

In line with our hypotheses H1 and H2, mothers were able to identify the BO of their own child and preferred this odour above the BO of unfamiliar children. HLA similarity had no major impact on the identification but did affect maternal preference of BOs. As predicted in H3, the preference for their own child's BO declined after infanthood but increased again after puberty. In line with H4, there was a higher preference for BO probes identified as the own child, irrespective of whether this identification was correct. Supporting H5, the maternal preference for the pubertal son's BO, but not for the BO of unfamiliar pubertal boys, decreased with increasing developmental status. In the following paragraphs, these results are discussed for each hypothesis and then converged in a combined model.

In terms of *kin recognition*, our data confirms previous studies [10,12,13] and demonstrates in a larger sample that healthy mothers can identify their own child's BO consistently throughout development, with the exception of early puberty. Although the identification ability of the present sample was above chance, it was worse compared to previous studies [49]. This might be owing to methodological differences (e.g. shorter exposure, more probes to choose, directly worn versus frozen BO samples). In our data, no HLA-related impact on kin recognition was found. Nevertheless, it is possible that a potential influence of genetics drives kin recognition directly after birth and that, over time, environmental compounds overrule this effect. Our study is not suited to test this hypothesis.

We suggest that *familiarity*—the feeling that one has smelled an odour before—contributes to olfactory kin recognition. In general, familiar odours, such as everyday life odours, are identified with higher likelihood [50] and preferred [51], and in the domain of BO, recognition of unrelated individuals is assumed to be mediated by familiarity [13,52]. The inability of mothers to identify their pubertal child fits this approach. During this developmental period, steroid hormones increase, which may consequently increase a child's BO intensity (e.g. [25]). Hence, the child does not smell familiar anymore. In the post-pubertal age group, this effect disappears as the ‘new’ BO becomes familiar again following prolonged exposure.

In terms of *preference*, mothers preferred the odour of their own child. The next most pleasant odour was the BO of an unfamiliar child who was in the same age group as the own child and HLA-similar to the mother. This supports previous results on the impact of HLA on BO perception [33,38,45] and implies that both developmental and genetic compounds contribute to BO preference. This effect is behaviourally relevant: a higher maternal preference for children with HLA-similar BOs might lead to higher parental investment in such children. This preference effect is reversed in the context of mate choice, where HLA dissimilarity was found to drive BO attractiveness ratings [37], in particular on HLA-B and -C [42], and such dissimilarity between mates enhances fitness of the offspring [34]. Age-related differences in maternal preference are discussed in the overall model below.

Maternal *identification behaviour* mediated the preference of the own child's odour. BO samples chosen as ‘own’ were strongly preferred over non-chosen probes, irrespective of whether they were the correct or false choice. Interestingly, this finding was most pronounced in the infant and pre-pubertal age group, supporting the idea that identification is a necessary antecedent for bonding, especially during the period where bonding is most crucial for the child [34,53]. The higher preference for chosen versus non-chosen BOs may function as one sensory mechanism enhancing action readiness, which facilitates a prompt reaction for the child's needs, as it has been reported for other modalities [4]. It has been further proposed that BOs function as *instrumental* and *affective* cues in the context of caregiving [16]. Where the instrumental value signals physiological needs (e.g. changing diapers), the affective value of BOs (e.g. reflected in pleasantness perception of the BO [15]) might function as a reward, promoting proximity to the child and, thus, driving emotional attachment. This might be particularly true for infancy and pre-pubertal ages, while pubertal children no longer depend on such intense parental care and attention. In this regard, bonding-related olfactory cues also lose their importance ([15], as compared with visual cues [14]) or change their meaning, e.g. signalling maturity over infantile needs.

According to H5, the *drop in BO ratings for pubertal sons* indicates that elevated testosterone levels during adolescence are expressed in BO and moderate the mother's perception. For daughters, it is unclear whether we observe the reverse effect: elevated oestradiol levels during puberty lead to an increase in pleasantness ratings. This might be owing to either a strong resemblance between the pubertal daughter's BO and the mother's own BO with regard to oestradiol level or to the fact that it is unclear whether oestradiol affects BO perception in a similar way to testosterone [25]. Oestradiol level of the donor has been shown to predict male ratings of women's BO attractiveness [30], and this might apply for female evaluation likewise. However, this was in the context of mate choice, whereas our study focused on the mother–child relationship, which is why context-dependent differences have to be considered. As testosterone and oestradiol concentrations fluctuate within an individual, we sampled the evening before the experimental night in order to assess the impact on BO. It is not yet understood in detail how chemical compounds released after hormonal changes affect BO composition, but our findings provide further support for their importance for social chemosignalling [19].

Pubertal status, referring mainly to visual characteristics, was naturally related to age and hormonal status. However, the impact of pubertal status on maternal ratings was not significant; we thus surmise that hormonal related olfactory cues do impact BO assessment. We speculate that testosterone functions as a potent olfactory cue, signalling maturity to the mothers. As children enter sexual maturity, inbreeding is possible but should be avoided. Therefore, BOs comprise a natural barrier to reduce the likelihood that mothers would mate with their own child (Westermarck effect [18]). In the two small experimental studies that exist to date, a mutual aversion of BO in father–daughter dyads in pubertal age but no such phenomenon in mother–son relationships was reported [13], and one study did not find any Westermarck-like effect at all [12]. Our data partly confirm the Westermarck effect, as we observed lower maternal liking for pubertal sons' odour compared to unfamiliar boys. However, incest avoidance does not fully explain our findings as maternal pleasantness evaluation dropped in the early pubertal stage but then recovered for late pubertal BOs. As discussed above, we speculate that these changes in pleasantness might be attributed to regained familiarity over time and, hence, regained pleasantness of son's odour.

We are aware of several limitations of this study. We assessed genetic and developmental compounds, operationalized with HLA and steroid hormones, but did not assess any other genetic compounds and hormones. Despite a rather large power of the overall sample, the sample size for the adolescent children was still small when split for sons and daughters, and not powerful enough to detect small effects. Furthermore, an investigation of the hormonal effects of daughters' BO on paternal perception would be enlightening. We still do not know whether fathers might be more prone to the Westermarck effect, as reported previously [13], although sibling-related studies demonstrate stronger inbreeding aversion for woman compared to men [54].

Taken together, we propose a model explaining maternal perception of their child's BO across age groups, which takes developmental, environmental and genetic compounds of BOs into account (see electronic supplementary material, figure S2). Maternal preference and identification of the own child's odour were *age-related* as they were observed for all groups except puberty. Differences between the age groups might be modulated by different factors that alter BO. Whereas BO preference relates to genetic similarity, kin recognition is based on familiarity, thereby overruling the genetic influence. Both mechanisms may facilitate targeted parental investment when bonding is most pivotal for a child. Developmental compounds play a large role in the critical period of puberty.

*In infants and pre-pubertal children*, kin recognition and preference of the own child's odour were stable. While kin recognition is presumably based on familiarity, HLA similarity may drive initial BO preference in these age groups as a potential mechanism of olfactory imprinting. The developmental influence of an infant's BO may relate to a perceived ‘cuteness’ of the BO in that age group, which mothers frequently report [15]. This is supported by the fact that mothers with impaired olfaction report a regret of the lack of that experience [55], and that this infantile smell activates reward areas in the maternal brain [56]. Infant cues elicit activations in hypothalamic, limbic and cortical networks mirroring parenting domains (for a review, see [57]). Infantile facial cuteness activates the pleasure network in mothers [4], indicating that similar effects can be triggered by olfactory cues when perceived as pleasant. Recruitment of this network relates to privileged processing and approach behaviour and thus evokes parental care [4].

*In puberty*, kin recognition and preference were not present. Both were presumably hindered by the dominant developmental influence of hormonal changes overriding genetics and familiarity: During this developmental stage, the own child's BO is not recognized because it does not smell familiar, and it is not preferred because it smells more intense. In addition, developmental compounds promote olfactory aversion of opposite-sex children, which may facilitate inbreeding avoidance.

*In post-pubertal children,* kin recognition and preference recovered. We assume that both improved as a function of increased familiarity, owing to a longer exposure to the BO and, hence, its evaluation as more pleasant again [51,52].

Further research into olfactory-mediated parental affection could investigate carers with either an impaired sense of smell or an impaired relationship to their child. Although we know that mothers with impaired bonding to their child do not prefer or recognize their child's odour [11], it is unclear whether their poor recognition abilities are a cause or a consequence. Future studies could focus on how differences in maternal behaviour contribute to differences in BO perception. In particular, maternal behaviour could be operationalized by an assessment of the relationship between observations of interpersonal touch or time spent in dyadic interaction and BO perception. The hedonic impact of familiar BOs demonstrates the possibility of clinical interventions supporting mother–child bonding, using olfaction as a promising target for such family interventions. Olfactory training or neurofeedback approaches using exposure to BO may thereby enhance sensory awareness, activate neural networks in response to pleasure, and, in turn, affect approach behaviour and parenting in a positive way.

## Supplementary Material

Supplementary material: Descriptive data and supplementary figure

## Data accessibility

The datasets supporting this article have been uploaded as part of the electronic supplementary material and are available from authors on request.
