# Supplementary material for: Body odours as a chemosignal in the mother–child relationship: new insights based on an human leucocyte antigen-genotyped family cohort
Source: Philos Trans R Soc Lond B Biol Sci. 2020 Apr 20;375(1800):20190266. doi: 10.1098/rstb.2019.0266 (PMC7209942; doi:10.1098/rstb.2019.0266)
Supplement: Supplementary material: Descriptive data and supplementary figure [file rstb20190266supp1.pdf]

PHILOSOPHICAL
TRANSACTIONS B

Supplement: Body odours as a chemosignal in the mother-child relationship: new insights based on an human leucocyte antigen-genotyped family cohort

Laura Schäfer[1*], Agnieszka Sorokowska[1,2], Jürgen Sauter[3], Alexander H. Schmidt[3,4], Ilona Croy[1]

[1†]Department of Psychosomatics, Technische Universität, Fetscherstr.74, 01307 Dresden, Germany, ORCID ID 0000-0002-6555-5854
[2]Smell and Taste Lab, Department of Psychology, University of Wroclaw, pl. Dawida 1, 50-527, Wroclaw, Poland
[3]DKMS, Kressbach 1, 72072 Tübingen, Germany
[4]DKMS Life Science Lab, St. Petersburger Straße 2, 01069 Dresden, Germany

Keywords: olfaction, parent-child relationship, body odor, MHC, hormones, chemosensory communication

Supplementary information on body odour matching (compare manuscript, method: BO matching):

For the own children, the mean similarity of shared HLA-B and -C alleles between mother and child across the whole sample was 2.2 alleles (SD = 0.53, ranging from 2 – 4 alleles, with 87% sharing 2 alleles, 8 % sharing 3 alleles, and 5 % sharing 4 alleles). For shared HLA-B and -C alleles between mothers and matched body odour (BO) samples across all HLA-similar unfamiliar children, the mean similarity was 1.75 alleles (SD = 0.62; for same aged children: M = 1.8, SD = 0.59, for the different aged children: M = 1.65, SD = 0.64; ranging from 1 to 4 alleles, with 36 % sharing 1 allele, 59 % sharing 2 alleles; 3 % sharing 3 and 2% sharing 4 alleles). Between mothers and matched BO samples across all HLA- dissimilar unfamiliar children, similarity was 0, as previously defined.

Supplementary tables

Supplementary Table 1. *Measures: Metrical variables displayed with M, SD, n for each age group and across all children.*

**variable**	**0-3 years**			**4-8 years**			**9-13 years**			**14-18 years**			**across all**		
	M	*SD*	*n*	*M*	*SD*	*n*	*M*	*SD*	*n*	*M*	*SD*	*n*	*M*	*SD*	*n*
number of children	1.42	.72	59	1.88	.64	40	2.09	.68	22	2.02	.60	43	1.79	.72	164
number of biological children	1.38	.56	59	1.82	.63	40	2.09	.68	22	1.98	.56	43	1.75	.65	164
smoking (years) in the past	2.46	1.95	14	2.98	2.81	12	.50	.71	2	1.17	1.47	3	2.30	2.25	34
BDI II	5.66	4.32	59	5.34	4.86	40	5.53	6.94	22	3.91	4.52	43	5.16	5.02	164
BIS score	21.11	3.15	59	22.12	3.32	40	21.41	3.22	22	19.49	3.01	43	21.03	3.28	164
BAS score	39.90	3.82	59	37.94	4.86	40	37.03	4.85	22	37.79	5.10	43	38.56	4.67	164
breastfeeding (in month)	10.45	7.42	52	11.23	6.10	33	11.42	5.38	16	8.35	7.27	30	10.41	6.76	164

Supplementary Table 2. *Measures: Categorical variables displayed with number, %, and n for each age group and across all children.*

**variable**	**category**	**0-3 years**			**4-8 years**			**9-13 years**			**14-18 years**			**across all**		
		number	*%*	*n*	*number*	*%*	*n*	*number*	*%*	*n*	*number*	*%*	*n*	*number*	*%*	*n*
educational degree	other/primary/ secondary school/ technical school /high school	2/ 0/ 14/ 4/ 39	1.9/ 0/ 24.5/ 7.5/ 66.0	59	0/ 1/ 13/ 0/ 26	0/ 2.9/ 32.4/ 0/ 64.7	40	0/ 0/ 6/ 2/ 14	0/ 0/ 27.3/ 9.1/ 63.6	22	0/ 0/ 14/ 3/ 26	0/ 0/ 32.6/ 7.0/ 60.5	43	1/ 1/ 44/ 11/ 106	.6/ .6/ 26.8/ 6.7/ 64.6	164
school-leaving qualification	apprentice/ completed apprenticeship/training/ technical school/ university of applied science/ University	2/ 6/ 19/ 3/ 3/ 27	1.9/ 9.4/ 32.1/ 5.7/ 5.7/ 45.3	59	2/ 7/ 8/ 2/ 2/ 19	5.0/ 17.5/ 20.0/ 5.0/ 5.0/ 47.5	40	0/ 4/ 7/ 3/ 2/ 6	0/ 18.2/ 31.8/ 13.6/ 9.1/ 27.3	22	0/ 9/ 7/ 8/ 4/ 15	0/ 20.9/ 16.3/ 18.6/ 9.3/ 34.9	43	3/ 25/ 39/ 16/ 11/ 69	1.8/ 15.2/ 23.8/ 9.7/ 6.6/ 42.1	164
university degree	Bachelor/ Magister/ Master/ Diploma/ Diploma university of applied science/ state exam/ phd	5/ 0/ 5/ 5/ 4/ 4/ 4	18.5/ 0/ 18.5/ 18.5/ 14.8/ 14.8/ 14.8	27	2/ 0/ 0 / 11/ 0/ 2/ 6	7.41/ 0/ 0/ 40.7/ 0/ 7.4/ 22.2	21	1/ 0/ 0/ 4/ 2/ 1/ 0	12.5/ 0/ 0/ 50.0/ 25.0/ 12.5/ 0	8	1/ 2/ 0/ 8/ 4/ 3/ 1	5.3/ 10.5/ 0/ 42.1/ 21.1/ 15.8/ 5.3	19	9/ 5/ 3/ 29/ 15/ 10/ 9	11.3/ 6.3/ 3.8/ 36.3/ 18.8/ 12.5/ 11.3	80
sexual orientation	hetero/ homo/ bisexual	57/ 2/ 1	96.6/ 3.4/ 1.7	59	39/ 1/ 0	97.5/ 2.5/ 0	40	22/ 0/ 0	100.0/ 0/ 0	22	42/ 0/ 1	97.7/ 0/ 2.3	43	159/ 3/ 2	96.7/ 2.0/ 1.3	164
relationship status	single/partnership with biological parent/ partnership with new partner	2/ 55/ 2	3.4/ 93.2/ 3.4	59	0/ 35/ 4	0/ 87.5/ 12.5	40	0/ 20/ 2	0/ 56.4/ 43.6	22	7/ 21/ 15	16.3/ 48.8/ 34.9	43	9/ 132/ 23	5.5/ 80.5/ 14.0	164
use of hormonal contraceptive	yes/ no	29/ 30	49.1/ 50.9	59	10/ 30	25/ 75	40	6/ 16	27.3/ 72.7	22	17/ 26	39.5/ 60.5	43	53/ 97	35.3/ 64.7	164
accident with head trauma	no/ yes	56/ 3	94.3/ 5.7	59	38/ 2	95.0/ 5.0	40	20/ 2	90.0/ 9.1	22	39/ 4	90.7/ 9.3	43	141/ 11	92.8/ 7.2	164
frequent sinusitis	no/ yes	59/ 0	100.0/ 0	59	37/ 3	91.2/ 8.8	40	21/ 1	95.5/ 4.5	22	41/ 2	95.3/ 4.7	43	146/ 6	96.1/ 3.9	164
hayfever	no/ yes	55/ 4	92.5/ 7.5	59	38/ 2	95.0/ 5.0	40	21/ 1	95.5/ 4.5	22	41/ 2	95.3/ 4.7	43	143/ 9	94.1/ 5.9	164
frequent cold	no/ yes	57/ 2	96.2/ 3.8	59	32/ 8	80.0/ 20.	40	22/ 0	100.0/ 0	22	40/ 3	93.0/ 7.0	43	139/ 13	91.4/ 8.6	164
obstructed nasal breathing	no/ yes	58/ 1	98.1/ 1.9	59	39/ 1	97.5/ 2.5	40	20/ 2	90.9/ 9.1	22	42/ 1	97.7/ 2.3	43	147/ 5	96.7/ 3.3	164
nervous disease	no/ yes	59/ 0	100.0/ 0	59	40/ 0	100.0/ 0	40	21/ 1	95.5/ 4.5	22	43/ 0	100.0/ 0	43	151/ 1	99.3/ .7	164
jaundice	no/ yes	59/ 0	100.0/ 0	59	40/ 0	100.0/ 0	40	22/ 0	100.0/ 0	22	43/ 0	100.0/ 0	43	152/ 0	100.0/ 0	164

diabetes mellitus	no/ yes	59/ 0	100.0/ 0	59	40/ 0	100.0/ 0	40	22/ 0	100.0/ 0	22	43/ 0	100.0/ 0	43	152/ 0	100.0/ 0	164
kidney disease	no/ yes	59/0	100.0/ 0	59	39/1	97.5/ 2.5	40	22/ 0	100.0/ 0	22	43/ 0	100.0/ 0	43	151/ 1	99.3/ 0.7	164
hyperthyroidism	no/ yes	58/ 1	98.1/ 1.9	59	39/1	97.5/ 2.5	40	21/ 1	95.5/ 4.5	22	43/ 0	100.0/ 0	43	149/ 3	98.0/ 2.0	164
hypothyroidism	no/ yes	48/ 11	79.2/ 20.8	59	35/ 5	87.5/ 12.5	40	19/ 3	86.4/ 13.6	22	37/ 6	86.0/ 14.0	43	127/ 25	83.6/ 16.4	164
parkinson´s disease	no/ yes	59/ 0	100.0/ 0	59	40/ 0	100.0/ 0	40	22/ 0	100.0/ 0	22	43/ 0	100.0/ 0	43	152/ 0	100.0/ 0	164
alzheimer´s disease	no/ yes	59/ 0	100.0/ 0	59	40/ 0	100.0/ 0	40	22/ 0	100.0/ 0	22	43/ 0	100.0/ 0	43	152/ 0	100.0/ 0	164
head surgery	no/ yes	54/ 5	90.2/9.8	59	38/ 2	95.0/ 5.0	40	22/ 0	100.0/ 0	22	43/ 0	100.0/ 0	43	152/ 0	100.0/ 0	164
paranasal sinuses	no/ yes	59/ 0	100.0/ 0	59	40/ 0	100.0/ 0	40	22/ 0	100.0/ 0	22	41/ 2	95.3/ 4.7	43	148/ 4	97.4/ 2.6	164
nasal septum	no/ yes	58/ 1	98.1/ 1.9	59	39/ 1	97.5/ 2.5	40	20/ 2	100.0/ 0	22	43/ 0	100.0/ 0	43	151/ 1	99.3/ .7	164
nasal conches	no/ yes	59/ 0	100.0/ 0	59	39/1	97.5/ 2.5	40	20/ 2	90.9/ 9.1	22	40/ 3	93.0/ 7.0	43	144/ 8	94.7/ 5.3	164
polyps	no/ yes	57/ 2	96.2/ 3.8	59	39/ 1	97.5/ 2.5	40	20/ 2	100.0/ 0	22	43/ 0	100.0/ 0	43	151/ 1	99.3/ .7	164
middle ear	no/ yes	58/1	98.1/ 1.9	59	40/ 0	100.0/ 0	40	20/ 0	100.0/0	22	43/0	100.0/ 0	43	152/0	100.0/0	164
Alcohol	yes/ no	27/ 32	45.1/ 54.9	59	18/ 22	45.0/ 55.0	40	13/ 9	59.1/ 40.9	22	29/ 14	67.4/ 32.6	43	80/ 69	53.7/ 46.3	164
Alcohol frequency	rarely/ medium/ frequently/ very frequently	22/ 5/ 0/ 0	82.6/ 17.4/ 0/ 0	27	10/ 7/ 1/ 0	55.6/ 38.9/ 5.6/ 0	18	6/ 3/ 3/ 1	46.2/ 23.1/ 23.1/ 7.7	13	18/ 7/ 3/ 1	62.1/ 24.1/ 10.3/ 3.4	43	51/ 19/ 7/ 2	64.6/ 24.1/ 8.9/ 2.5	79
current smoking	no/ not anymore / occasionally/ regularly	36/ 16/ 6/ 1	60.8/ 27.5/ 9.8/ 2.0	59	17/ 15/ 8	42.5/ 37.5/ 20.0	40	17/ 2/ 1/ 2	77.3/ 9.1/ 4.5/ 9.1	22	32/ 6/ 4/ 1	74.4/ 14.0/ 9.3/ 2.3	43	102/ 38/ 20/ 4	62.8/ 23.0/ 12.2/2.3	164
exposure to chemicals	yes/ no	4/55	7.8/ 92.2	59	0/ 40	0/ 100.0	40	1/ 21	4.5/ 95.5	22	1/ 42	2.3/ 97.7	43	6/ 164	3.7/96.3	164
preterm birth	yes/ no	5/ 54	6.8/ 93.2	59	7/ 44	13.7/ 86.3	44	2/ 32	5.9/ 94.1	34	6/ 41	12.8/ 87.2	47	20/ 186	4.1/ 95.9	206
knowledge of the child´s body odour	yes/ no	54/ 5	91.9/ 8.1	59	49/ 2	96.1/3.9	51	29/ 5	85.3/ 14.7	34	42/ 5	89.4/ 10.6	47	188/ 18	91.3/ 8.7	206
growth of pubic hair	not begun/ slowly begun/ definetly begun/ adult	59/ 0/ 0/0	100/ 0/ 0/ 0	59	38/ 1/ 3/ 4	82.6/ 2.2/ 6.5/ 8.7	45	12/ 1/ 11/ 10	35.3/ 2.9/ 32.4/ 29.4	19	1/ 1/ 11/ 34	2.1/ 2.1/ 23.4/ 72.3	47	70/ 3/ 26/ 49	47.3/ 2.0/ 17.6/ 33.1	148
breast growth	not begun/ slowly begun/ definetly begun/ adult	59/ 0/ 0/ 0	100/ 0/ 0/ 0	59	14/ 5/ 2/ 2	60.9/ 21.7/ 8.7/ 8.7	23	3/ 4/ 9/ 3	15.8/ 21.1/ 47.4/ 15.8	15	0/ 0/ 7/ 17	0/ 0/ 29.2/ 70.8	24	26/ 10/ 19/ 22	33.8/ 13.0/ 24.7/ 28.6	77
beard growth	not begun/ slowly begun/ definetly begun/ adult	59/ 0/ 0/ 0	100/ 0/ 0/ 0	59	22/ 0/ 0/ 1	95.7/ 0/ 0/ 4.3	23	9/ 1/ 1/ 4	60.0/ 6.7/ 6.7/ 26.7	19	3/ 5/ 6/ 9	13.0/ 21.7/ 26.1/ 39.1	23	43/ 6/ 8/ 14	60.6/ 8.5/ 11.3/ 19.7	71
menstruation	no/ yes	59/ 0	100/0	59	19/ 4	82.6/ 17.4	23	8/ 11	42.1/ 57.9	19	0/ 24	0/ 100.0	24	37/ 40	48.1/ 51.9	77
voice break	not begun/ slowly begun/ definitely begun/ adult	0/ 0/ 0/ 0	100/ 0/ 0/ 0		21/ 1/ 0/ 1	91.3/ 4.3/ 0/ 4.3	23	6/ 3/ 1/ 5	40.0/ 20.0/ 6.7/ 33.3	15	2/ 3/ 2/ 16	8.7/ 13.0/ 8.7/ 69.6	23	38/ 8/ 3/ 22	53.5/ 11.3/ 4.2/ 31.0	71

Supplementary Table 3. *Descriptive values of pleasantness ratings (M, SE, SD, CI) depicted for each BO sample for each age group; and for BOs chosen as "own child" vs. non-chosen as "own child".*

**BO**	**age group**	across all BOs				BOs chosen as "own child"				BOs not chosen as "own child"			
		M	*SE*	*SD*	*CI*	*M*	*SE*	*SD*	*CI*	*M*	*SE*	*SD*	*CI*
own child	0-3	64.82	2.89	24.85	[59.07; 70.58]	76.63	4.58	23.79	[67.22; 86.04]	58.04	3.36	23.05	[51.27; 64.81]
	4-8	65.46	3.32	23.91	[58.80; 72.12]	70.39	4.48	18.99	[60.94; 79.83]	62.85	4.46	26.02	[53.77; 71.93]
	9-13	60.40	3.62	23.44	[53.10; 67.71]	66.18	6.20	20.56	[52.37; 79.99]	58.35	4.37	24.36	[49.42; 67.29]
	14-18	65.61	2.97	21.83	[59.65; 71.57]	66.45	4.64	21.75	[56.81; 76.10]	65.03	3.93	22.21	[57.02; 73.04]
	across all	64.43	1.58	23.57	[61.21; 67.45]	60.80	1.89	23.82	[56.87; 64,47]	70.85	2.47	21.79	[65.89; 75.76]
HLA similar same age group	0-3	64.04	2.76	23.95	[58.53; 69.55]	75.45	6.03	20.01	[62.01; 88.90]	62.08	3.02	24.15	[56.04; 68.11]
	4-8	59.65	3.82	27.52	[51.99; 67.32]	54.75	8.56	29.66	[35.91; 73.59]	61.12	4.28	27.07	[52.47; 69.78]
	9-13	61.42	3.93	24.45	[53.45; 69.39]	63.67	12.98	22.48	[7.82; 119.51]	61.23	4.17	24.69	[52.75; 69.71]
	14-18	55.28	3.48	25.34	[48.30; 62.27]	69.50	7.14	17.48	[51.16; 87.84]	53.47	3.75	25.74	[45.91; 61.03]
	across all	60.41	1.71	25.27	[57.03; 63.78]	59.54	1.85	25.34	[55.87;63.20]	65.47	4.35	24.62	[56,59; 74.35]
HLA dissimilar same age group	0-3	57.99	2.75	24.61	[52.52; 58.29]	72.00	4.38	16.39	[62.53; 81.46]	55.02	3.09	25.12	[48.84; 61.19]
	4-8	57.06	3.41	25.08	[50.21; 63.90]	72.62	5.96	16.85	[58.54; 86.71]	54.35	3.75	25.42	[46.80; 61.90]
	9-13	53.38	4.08	24.85	[45.09; 61.66]	56.17	9.21	22.56	[32.49; 79.84]	52.84	4.59	25.58	[43.46; 62.22]
	14-18	59.33	3.01	22.35	[53.28; 65.37]	60.00	6.62	19.85	[44.74; 75.26]	59.20	3.39	23.01	[52.36; 66.03]
	across all	57.33	1.61	24.15	[54.17; 60.50]	55.51	1.79	24.67	[51.97;59.05]	66.65	3.11	18.94	[60.33;72.96]
HLA similar different age group	0-3	56.96	2.23	21.24	[52.52; 61,38]	79.33	10.40	18.01	[34.60; 96.07]	56.19	2.24	21.00	[51.74; 60.64]
	4-8	58.12	3.12	23.16	[51.85; 64.37]	72.33	10.10	24.74	[46.36; 98.30]	56.37	3.23	22.62	[49.87; 62.86]
	9-13	58.45	4.12	26.68	[50.14; 66.77]	64.00	15.53	26.91	[2.84; 93.84]	58.02	4.32	26.97	[49.28; 66.77]
	14-18	54.72	4.45	26.69	[45.69; 63.75]	54.80	14.68	32.83	[14.04; 95.56	54.71	4.71	26.22	[45.09; 64.33]
	across all	57.16	1.57	23.58	[45.05; 60.26]	56.36	1.61	23.25	[53.18;59.54]]	66.96	6.32	26.06	[53.54;80.43]
HLA dissimilar different age group	0-3	57.34	2.57	24.63	[52.24; 62,44]	72.67	5.23	12.82	[59.22; 86.12]	56.27	2.69	24.94	[50.92; 61.62]
	4-8	56.08	3.53	25.24	[48.98; 63.18]	66.40	11.56	25.85	[34.30; 98.50]	54.96	3.72	25.21	[47.47; 62.44]
	9-13	52.32	3.72	24.65	[44.82; 59.81]	69.50	2.18	4.36	[62.56; 76.44]	50.60	3.98	25.20	[42.54; 58.66]
	14-18	49.64	3.86	24.08	[41.83; 57.45]	51.43	12.21	32.29	[21.56; 81.30]	49.25	3.98	22.54	[41.12; 57.38]
	across all	54.57	1.64	24.69	[51.51; 55.33]	53.75	1.72	24.63	[50.35; 57.17]	63.91	4.99	23.41	[53.5; 74.29]

Note. *Abbreviations: BO = body odour*

Bayesian analyses (compare manuscript, method: statistical analyses)

In order to additionally explore the likelihood of our hypotheses, we used Bayesian statistics [1] using JASP software [2]. We included only H1 and the first assumption of H2 for that analysis, as for these hypotheses basic assumptions about the effect sizes are available based on a previous publication [3].

H1: Mothers are able to identify their own child above chance

We performed a Bayesian Binomial test using the identification of own child (yes/no) as the dependent variable:

Based on a previous study [3] we assumed that the identification ability for the choice between three BO samples is at 75%; and rescaled this to our design with a choice between six BO samples, = 37.5%. We, therefore, did not follow the suggestions from Dienes & Mclatchie (2018, [1]) to divide the value by 2, because the result would then equal the chance level (null hypothesis = correct identification of child does not differ from chance level, chance level = 16.67 %). We

hence tested evidence for the alternative hypothesis (correct identification of own child above chance) against 0.375. Additionally, we tested evidence for the null hypothesis against 0.167. We did this for each age group, as listed in the Table below.

Supplementary Table 4. *Bayesian Factor (BF) for own child identification comparing null and alternative hypothesis*.

age group	test against 0.167	test against 0.375	results in favor of
0-3	BF <0.001	BF 6.7	alternative hypothesis
4-8	BF 0.016	BF 6.0	alternative hypothesis
9-13	BF 1.27	BF 2.2	neither alternative nor null hypothesis
14-18	BF <0.001	BF 4.6	alternative hypothesis

The Table shows a Bayesian Factor (BF) below 3 for all groups in comparison to the null hypothesis, indicating that the null hypothesis is not supported in any of the groups. In addition, the BF is higher 3 for all groups except for age group 9-13, indicating that the alternative hypothesis is supported for those groups. For age group 9-13, neither the alternative hypothesis nor the null hypothesis are supported by the Bayesian analyses.

The interpretation of the BF was guided by Dienes & Mclatchie, 2018 [1] who referred to Jeffrey (1998, [4]), in interpreting a BF of >3 as in favor of the alternative hypothesis.

H2: Mothers prefer the BO of their own child over other children

We performed the Bayesian Factor for a one sample t-test using the preference of the own vs. unfamiliar child as the dependent variable. Based on a previous study [3] we assumed that the difference in pleasantness ratings between the own and unfamiliar children´s BO is 1.3 points (pleasantness ratings on a scale ranging from 1 to 10); and rescaled this to our design (pleasantness ratings on a scale ranging from 1 to 100) thereby expecting a difference of 13 points. According to suggestions from Dienes & Mclatchie (2018), we divided this value by 2; thus, we tested against 6.5.

Again, we tested evidence for the alternative hypotheses (significant difference between pleasantness ratings for own vs. unfamiliar children) against 6.5, and additionally, we tested null hypothesis (no difference between own and unfamiliar children) against 0. We performed the analyses for each age group. Results are presented in the Table below.

Supplementary Table 5. *Bayesian Factor (BF) for own child preference comparing null and alternative hypothesis.*

age group	test against 0	test against 6.5	results in favor of
0-3	BF 0.7	BF 7.7	alternative hypothesis
4-8	BF 0.4	BF 5.6	alternative hypothesis
9-13	BF 7.8	BF 2.9	alternative hypothesis rejected
14-18	BF 0.06	BF 3.4	alternative hypothesis

The Table shows a BF below 3 for all groups except of age group 9-13 in comparison to the null hypothesis, indicating that the null hypothesis is not supported for three groups, but supported for 9-13 year old children. In addition, the BF is higher 3 for all groups except for age group 9-13, indicating that the alternative hypothesis is supported for those groups. For age group 9-13 the null hypothesis is supported, meaning that there is no pleasantness preference of the own child´s BO compared to unfamiliar children.

Supplementary figures

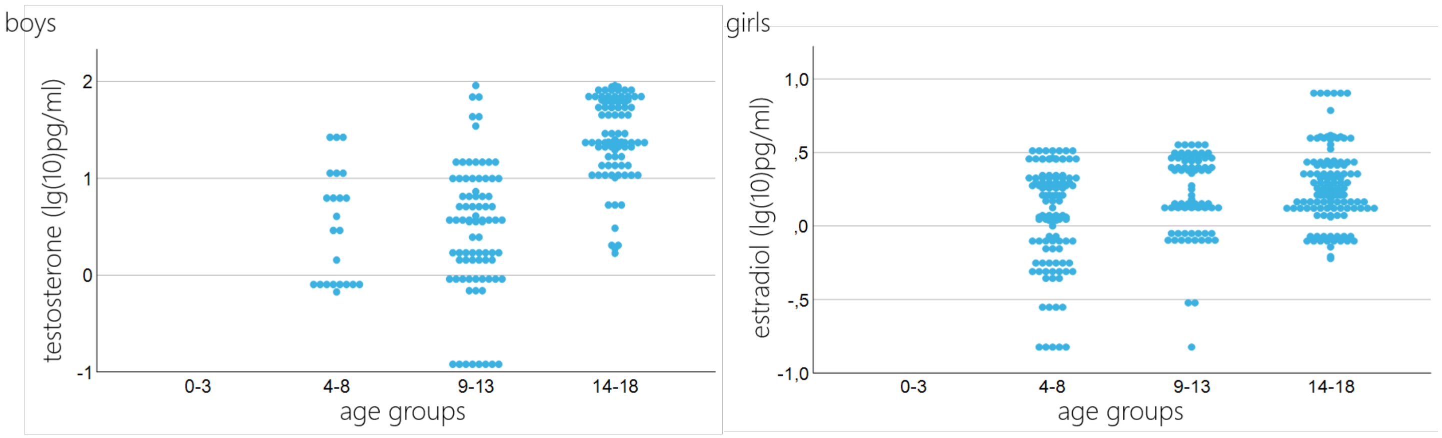

Supplementary Figure 1. Hormonal concentration in relation to child´s age group. Hormonal concentration depicted as lg(10), pg/ml. Left: testosterone (boys); right: estradiol (girls).

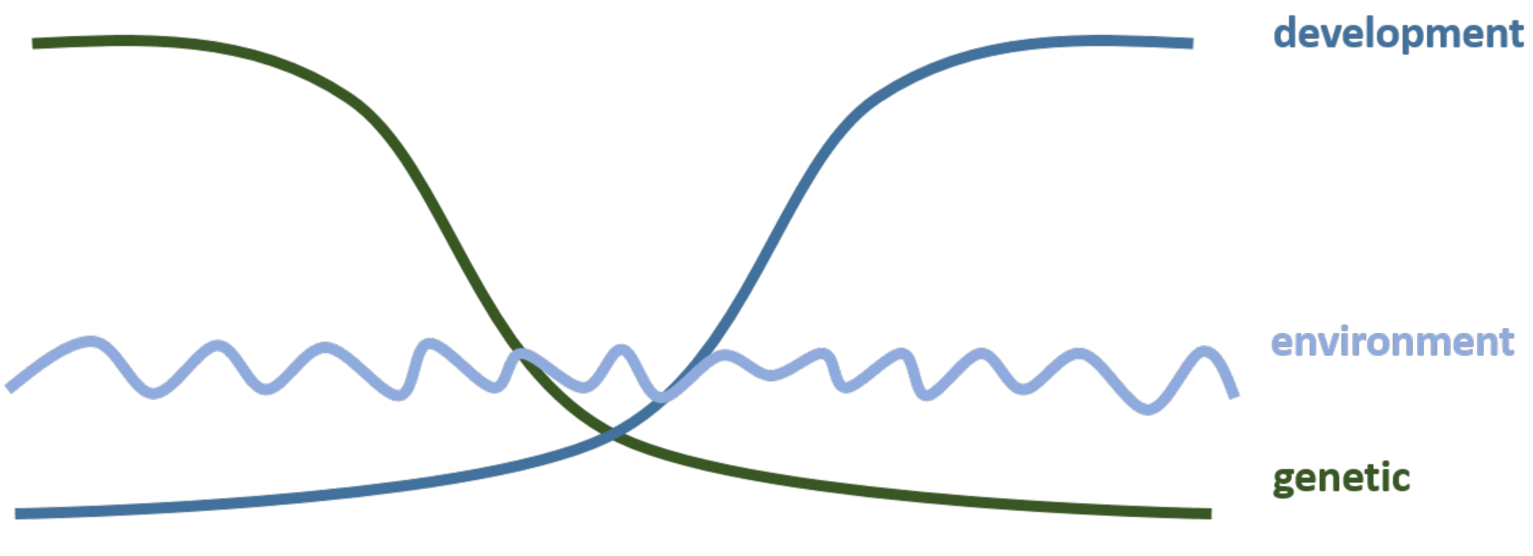

Supplementary Figure 2. Schematic visualization of factors contributing to maternal pleasantness perception of their child´s BO in relation to age.

References

1. Dienes, Z. and N. Mclatchie, *Four reasons to prefer Bayesian analyses over significance testing.* Psychonomic bulletin & review, 2018. **25**(1): p. 207-218.
2. Team, J., *JASP (version 0.9)[computer software]*. 2018.
3. Croy, I., et al., *Mother-child bonding is associated with the maternal perception of the child's body odor.* Physiology & behavior, 2019. **198**: p. 151-157.
4. Jeffreys, H., *The theory of probability*. 1998: OUP Oxford.
